# Neurophysiological mechanisms underlying cardiovascular adaptations to exercise: A narrative review

**DOI:** 10.14814/phy2.70439

**Published:** 2025-07-09

**Authors:** Joseph G. Omole, Idara A. Okon, Godswill J. Udom, Omoirri M. Aziakpono, Richard D. Agbana, Ayodeji Aturamu, Nicodemus Niwamanya, Benjamin Oritsemuelebi, Ekom M. Etukudo, Omoniyi K. Yemitan

**Affiliations:** ^1^ Department of Physiological Sciences, Faculty of Basic Medical Sciences Obafemi Awolowo University Ile‐Ife Nigeria; ^2^ Department of Physiology, Faculty of Biomedical Sciences Kampala International University Ishaka‐Bushenyi Uganda; ^3^ Department of Pharmacology and Toxicology, Faculty of Pharmacy Federal University Oye‐Ekiti Ekiti Nigeria; ^4^ Department of Pharmacology and Toxicology, School of Pharmacy Kampala International University Ishaka‐Bushenyi Uganda; ^5^ Department of Pharmacology, Faculty of Pharmaceutical Sciences and Medicine Kampala International University Gongolamboto‐Dar es Salaam Tanzania; ^6^ Department of Community Medicine, College of Medicine and Health Sciences Afe‐Babalola University Ado‐Ekiti Nigeria; ^7^ Department of Medical Physiology, College of Medicine Ekiti State University Ado‐Ekiti Nigeria; ^8^ Department of Pharmacology and Therapeutics Delta State University Nigeria; ^9^ Department of Anatomy, Faculty of Biomedical Sciences Kampala International University Ishaka‐Bushenyi Uganda; ^10^ Department of Pharmacology, Therapeutics and Toxicology Lagos State University College of Medicine Ikeja Nigeria

**Keywords:** brain–heart axis, multimodal exercise interventions, neurocardiogenic regulation, neuroplasticity

## Abstract

The brain–heart connection, particularly during physical activity, plays a crucial role in health and disease management. This review examined the neurophysiological mechanisms driving cardiovascular adaptations to exercise, focusing on the bidirectional relationship between the brain and heart. Key mediators such as central autonomic networks, brain‐derived neurotrophic factors (BDNF), and vascular endothelial growth factor (VEGF) enhance neural plasticity and vascular health. Regular structured exercise (e.g., high‐intensity interval training, moderate and resistance exercise) moderates autonomic responses, increases BDNF, and supports neurovascular coupling, improving both cognitive and cardiovascular resilience through molecular pathways such as PGC‐1α and TrkB signaling. Exercise enhances cerebral perfusion, reduces oxidative stress, and protects brain–heart health. It mitigates risks linked to neurodegenerative diseases, such as Alzheimer's and Parkinson's, by promoting neuroplasticity and vascular integrity. This review highlights the importance of incorporating exercise‐based interventions in clinical practice and public health policies to optimize cognitive and cardiovascular health. Future studies should explore exercise‐induced neurovascular coupling to further elucidate the mechanisms connecting brain and cardiovascular health.

## Graphical abstract

Cardiovascular adaptations during exercise.

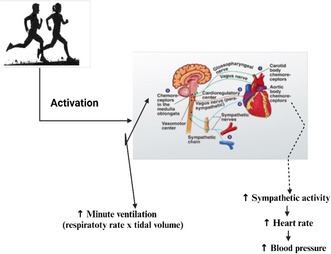

## 1 INTRODUCTION

The brain–heart connection is a critical component of physiological regulation, mediated by intricate autonomic and endocrine pathways (Liu et al., 2022). This bidirectional interaction has gained increasing scientific attention, particularly due to its role in conditions like stroke, cardiovascular diseases, and lifestyle‐related disorders. Neurocardiogenic mechanisms, including vasovagal responses characterized by abrupt autonomic dysregulation and altered vascular tone, are implicated in approximately 1.5 million global deaths annually (Liang et al., 2024; Manolis et al., 2021). Sedentary lifestyles and post‐stroke complications further amplify this risk (Dhondt et al., 2021).

The autonomic nervous system (ANS), limbic circuitry, and renin–angiotensin–aldosterone system collaboratively modulate the heart–brain axis, representing therapeutic targets for neurological and cardiovascular disorders (Elia & Fossati, 2023). The central autonomic network (CAN), comprising the prefrontal cortex, anterior cingulate, insular cortex, amygdala, hypothalamus, periaqueductal gray, and brainstem nuclei, regulates cardiac output, vascular tone, and sympathetic activation (Lamotte et al., 2021). The hypothalamus is pivotal in exercise‐induced homeostasis, modulating mood, hormonal responses, and cardiovascular dynamics (Bliss et al., 2023). Regular exercise enhances neuroplasticity, autonomic balance, metabolic efficiency, and antioxidant defenses, providing neuroprotective and cardioprotective effects (da Silva et al., 2022; Hu et al., 2023). These adaptations improve oxygen delivery, tissue perfusion, and motor control, while reducing neurocardiogenic dysfunction risks (de Sousa Fernandes et al., 2020). Neurocardiogenic dysfunction contributes to stroke, cardiac arrhythmias, and metabolic disorders. Preclinical and clinical studies highlight exercise's role in mitigating neural, muscular, and cognitive impairments (Marques‐Aleixo et al., 2021; Orsini et al., 2018). Amid increasing sedentary lifestyles, understanding molecular and neurophysiological mechanisms linking exercise, brain function, and cardiovascular health is crucial. Therefore, this review explores exercise's neurophysiological mechanisms underlying cardiovascular adaptations and their health implications.

## 2 NEUROPHYSIOLOGICAL BASIS OF CARDIOVASCULAR CONTROL DURING EXERCISE

The heart‐brain axis represents a complex network of autonomic and central nervous system circuits coordinating cardiovascular regulation (Mohanta et al., 2023). Cardiac sensory input is transmitted by sympathetic afferent fibers through the dorsal root ganglia and spinal cord to the nucleus tractus solitarius (NTS) in the medulla oblongata (Fang & Zhang, 2024). Concurrently, parasympathetic signals travel via the vagus nerve to the dorsal motor nucleus and nucleus ambiguus, converging at the NTS before projecting to higher brain centers such as the cerebral cortex (Forstenpointner et al., 2022; Neuhuber & Berthoud, 2022). Within the CAN, these signals orchestrate sympathetic and parasympathetic outputs (Valenza et al., 2024). Baroreflex, a key regulatory mechanism, maintains blood pressure homeostasis by adjusting heart rate, vascular tone, and stroke volume (Herring et al., 2024). Exercise upregulates sympathetic activity through CAN‐mediated noradrenaline release on β1‐adrenergic receptors (Priel et al., 2021), whereas parasympathetic dominance during rest reduces heart rate via muscarinic receptor activation (Fedele & Brand, 2020). Emerging studies highlight cortical modulation of heart rate variability (Guendelman et al., 2024; Schumann et al., 2021). Physical activity reduces sympathetic tone by modulating the rostral ventrolateral medulla, while inactivity enhances sympathetic drive and cardiovascular risk (Guyenet & Stornetta, 2022; Kulkarni et al., 2023; Hafez & Chang, 2025).

### 2.1 Cortical influence on cardiovascular control

The cerebral cortex, particularly motor‐related regions, modulates cardiovascular functions such as heart rate and blood pressure, although autonomic control remains dominant (Karim et al., 2023; Skolasinska et al., 2023). Cortical influence becomes prominent in response to emotional, psychological, and environmental cues (Vaccarino et al., 2021). For example, the prefrontal cortex integrates emotional stimuli—stress, excitement, and anxiety—which can increase blood pressure through sympathetic activation (Esler, 2022). Baroreceptor input is processed in the brainstem to adjust cardiovascular output, but cortical modulation can refine this response (Hafez & Chang, 2025). Recent findings show that cortical regions can exert voluntary control over cardiovascular function during physical activity (Zheng et al., 2025). Electroencephalogram studies reveal that moderate exercise activates the primary motor cortex, while excessive intensity may suppress cerebellar function, impairing coordination despite increased cardiac output (Fontes et al., 2020). The prefrontal cortex governs attention and cognition during exercise (Robertson & Marino, 2016), with subregions like the dorsolateral and orbitofrontal cortices supporting memory and decision‐making (Jobson et al., 2021). However, high‐intensity exercise may impair prefrontal activity and cognitive function (Komiyama et al., 2020), while low‐to‐moderate aerobic exercise enhances cortical activity and improves vascular health (Zhang, Zhang, et al., 2021). The insular cortex, particularly the left side, integrates interoceptive signals and autonomic control, helping regulate cardiovascular responses to physical demand (Karim et al., 2023; Ramos et al., 2024). The interaction between the prefrontal and insular cortices illustrates how cortical regions shape cardiovascular adaptation during exercise (Bigliassi, 2022; Fontes et al., 2020).

## 3 BRAIN‐DERIVED NEUROTROPHIC FACTOR AND CARDIOVASCULAR HEALTH

### 3.1 Exercise‐induced neural plasticity

**FIGURE 1:**
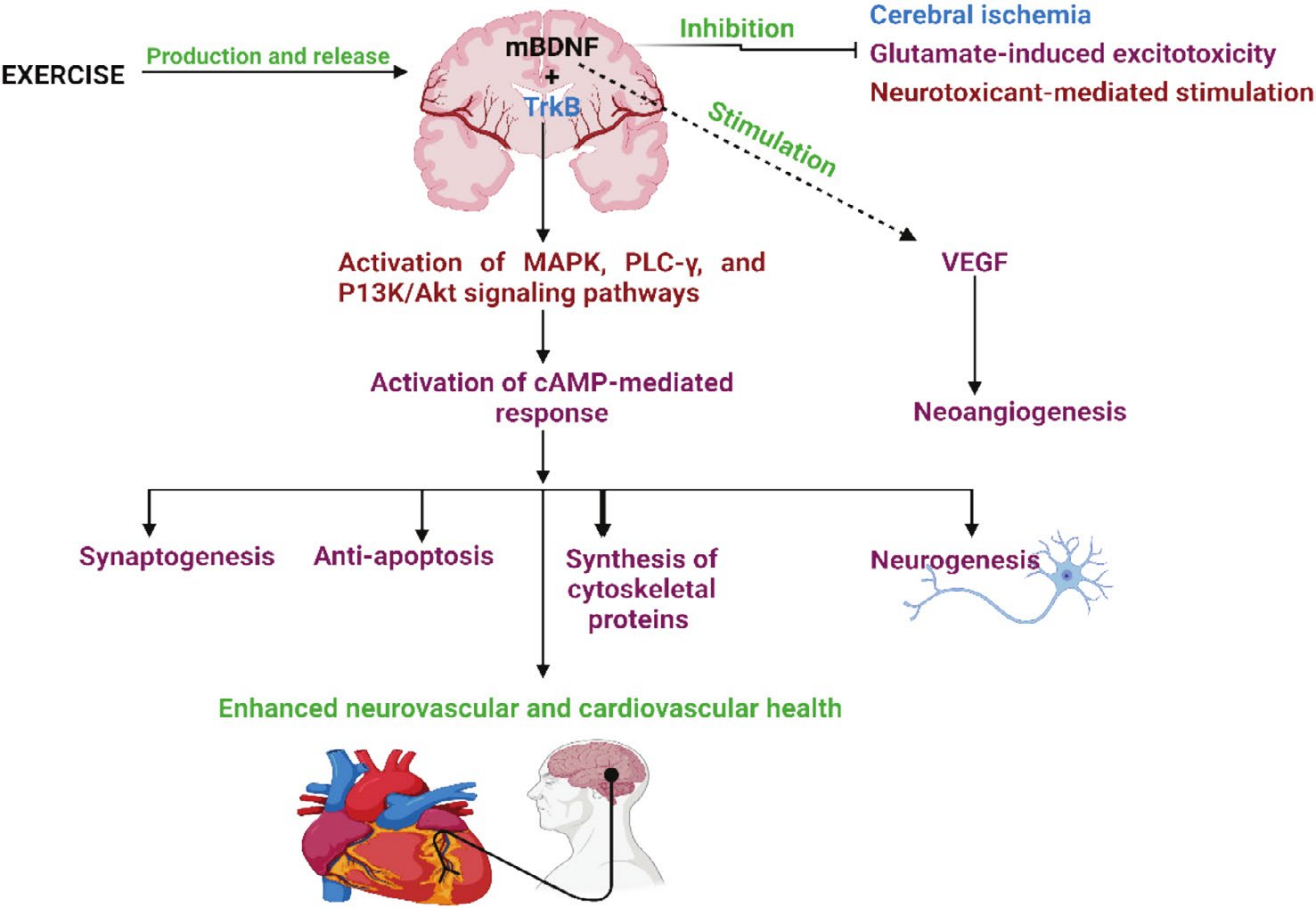
Molecular mechanisms for BDNF neuroprotection. Akt, Akt enzyme or protein kinase B; MAPK, mitogen‐activated protein kinase; mBDNF, mature brain‐derived neurotrophic factor; P13K, phosphatidylinositol 3‐kinase; PLC‐γ, phospholipase C‐γ; TrKB, tropomyosin receptor kinase; VEGF, vascular endothelial growth factor.

Exercise enhances neural plasticity, particularly within autonomic regulatory centers, thereby improving cardiovascular function (Figure 1). It stimulates synaptic plasticity and upregulates neurotrophic factors, notably brain‐derived neurotrophic factor (BDNF) and lactate, which support neuronal survival and synaptic connectivity (Daniela et al., 2022; Müller et al., 2020). BDNF (a neurotrophin produced by neurons and glial cells) plays a critical role in neuronal growth, differentiation, survival, and synaptic modulation through activation of its receptor, tropomyosin receptor kinase B (TrkB) (Costa et al., 2022). This activation triggers key signaling cascades such as PI3K/Akt, MAPK/ERK, and PLCγ, essential for neuroplasticity and cognitive function (Bazzari & Bazzari, 2022). Additionally, BDNF modulates neuroinflammation by suppressing proinflammatory cytokines (e.g., TNF‐α, IL‐1β) and enhancing anti‐inflammatory responses, including IL‐4 production (Boakye et al., 2021). It also supports neuronal survival through apoptosis inhibition and axon myelination (Schirò et al., 2022), with both aerobic and anaerobic exercise influencing its release (Murawska‐Ciałowicz, de Assis, et al., 2021). Exercise‐induced BDNF expression is further regulated by factors like fasting, hypoxia, and diet via the BDNF/VEGF/PI3K pathway (Katare et al., 2009). Neuroplastic changes in the hippocampus and prefrontal cortex following exercise enhance cognition and autonomic regulation of cardiovascular parameters such as blood pressure and heart rate (Farì et al., 2021; Sadri et al., 2024; Wang et al., 2020). Epigenetically, exercise influences microRNAs such as miR‐132 and miR‐34c implicated in synaptic remodeling and memory (Carvalho et al., 2021). Furthermore, exercise enhances mitochondrial biogenesis via PGC‐1α and Nrf2 pathways, promoting oxidative resilience, and increases VEGF and IGF‐1, vital for neurogenesis and vascular health (Arjunan et al., 2023; Zorina et al., 2023).

### 3.2 Vascular endothelial growth factor and angiogenesis in the brain and heart

Vascular endothelial growth factor (VEGF) plays a crucial role in exercise‐induced angiogenesis, enhancing vascular plasticity and improving cerebral and cardiac blood flow (Hashimoto et al., 2021). In the brain, vascular endothelial growth factor supports endothelial cell health, maintaining the blood–brain barrier and cerebral circulation. The vascular endothelium plays a critical role in cardiovascular health, and its function is closely tied to brain regulation (Bkaily & Jacques, 2023). Exercise also increases nitric oxide production, a vasodilator that reduces oxidative stress via the eNOS/NO/cGMP pathway, improving vascular tone and microcirculatory function (De Ciuceis et al., 2023; Woloschuk et al., 2020). Vascular tone during exercise is largely determined by the eNOS/NO/cGMP mechanism (Tran et al., 2022). The function of this vascular control mechanism influences exercise tolerance and its benefits. Therefore, neurophysiological signaling between the brain and cardiovascular system further optimizes blood vessel dilation, reinforcing the cardiovascular benefits of regular exercise (Claassen et al., 2021).

## 4 NEUROPHYSIOLOGICAL IMPACT OF HIGH‐INTENSITY INTERVAL TRAINING ON CARDIO‐CEREBROVASCULAR HEALTH

High‐intensity interval training (HIIT) has emerged as an efficient strategy for enhancing both cardiovascular and cognitive health in non‐athletes (Wang et al., 2023). In a study involving 20 healthy, sedentary males, HIIT significantly reduced systolic blood pressure without inducing structural or functional cardiac alterations (Holloway et al., 2018). Comparatively, HIIT demonstrated superior efficacy over moderate continuous training in improving brachial artery flow‐mediated dilation and decreasing aortic pulse wave velocity in physically inactive adults (Ramírez‐Vélez et al., 2019). HIIT elevates BDNF and stimulates mitochondrial biogenesis in brain and muscle tissues, enhancing cognitive function and cardiovascular resilience (Hu et al., 2021). Emerging evidence indicates that HIIT may provide equal or superior cognitive benefits compared to moderate‐intensity exercise (Mekari et al., 2020). In stroke rehabilitation, low‐volume HIIT improves gait in chronic stroke patients, while long‐interval HIIT benefits high‐functioning individuals (Crozier et al., 2018). Short‐interval HIIT increases ventilatory thresholds and walking speed, while higher intensities enhance neuroplasticity and motor skill retention (Beck et al., 2020; Luo et al., 2019; Nepveu et al., 2017; Palmer et al., 2024). Differential effects on BDNF/TrkB signaling have been observed: short HIIT increases mature BDNF, while longer intervals elevate pTrkB/TrkB ratios (Hugues et al., 2023). During stroke recovery, HIIT may influence BDNF and IGF‐1 more than VEGF (Górna et al., 2025). Moreover, HIIT improves cerebral blood flow, oxygenation, and reduces oxidative stress in models of vascular dementia (Guo et al., 2023; Puoyan‐Majd et al., 2024). Although direct prevention of microinfarcts remains unproven (Smith et al., 2012), HIIT may mitigate risk through improved cardiovascular fitness and reduced comorbidities such as obesity and hypertension (Twerenbold et al., 2023).

### 4.1 HIIT, BDNF, and cardiovascular resilience

HIIT acutely elevates circulating blood BDNF levels (Murawska‐Ciałowicz, de Assis, et al., 2021). The release of BDNF during HIIT improves neurovascular coupling, facilitating more efficient regulation of cerebral blood flow in response to neural activity (Erickson et al., 2022). Elevated BDNF levels are associated with improved endothelial function and reduced cardiovascular risk (Fioranelli et al., 2023; Hang et al., 2021). Higher exercise intensity improves cardiovascular health and survival against risk factors in young, healthy individuals (Okon, Beshel, et al., 2024). Both acute and chronic HIIT interventions have been shown to increase BDNF production, thereby enhancing mood and cognitive performance (Tsai et al., 2021). BDNF further supports the brain–heart axis by promoting angiogenesis, neuronal survival, and cardiovascular regulation (Pius‐Sadowska & Machaliński, 2017; Salmina et al., 2021; Trombetta et al., 2020).

Cardiovascular disease (CVD) is predominantly driven by atherosclerosis, a chronic inflammatory condition initiated by endothelial dysfunction and activation (Frąk et al., 2022). This pathological process involves vasoconstriction, persistent inflammation, and the development of atheromatous plaques within arterial walls (Henein et al., 2022). BDNF has been shown to play a crucial role in cardiovascular regulation. Reduced circulating BDNF levels are linked to impaired endothelial and vascular function, a phenomenon exacerbated by elevated oxidative stress (Jin et al., 2021). In individuals with heart failure, diminished BDNF concentrations correlate with heightened cardiovascular risk (Pius‐Sadowska & Machaliński, 2017). Several established cardiovascular risk factors, including hyperlipidemia, advanced age, male sex, smoking, and diabetes, are associated with lower BDNF levels (Levinger et al., 2008). Moreover, BDNF deficiency may contribute to endothelial cell dysfunction, serving as a potential predictor of adverse cardiovascular outcomes (Trombetta et al., 2020). The interplay between endothelial injury and reduced BDNF may be bidirectional, with each exacerbating the other and impairing endothelial cell survival and angiogenesis (Ma et al., 2021). Notably, HIIT has been shown to elevate both central and peripheral BDNF levels, thereby enhancing cardiovascular function through its beneficial vascular effects (García‐Suárez et al., 2021).

## 5 NEUROVASCULAR ADAPTATIONS TO EXERCISE

The brain's regulation of cerebral blood flow (CBF) involves neurovascular coupling, cerebral perfusion pressure, humoral factors, cardiac output, and neuronal activity through action potentials and synapses (Claassen et al., 2021). Exercise enhances brain structure and function, with habitual exercise enlarging the hippocampus and prefrontal cortex (Zhang, Zong, et al., 2021), increasing blood and oxygen delivery, stimulating neuron growth, and strengthening synaptic connections. Exercise preferentially increases cerebral blood volume in the occipital regions compared to the temporal and frontal lobes (Foster et al., 2020), with CBF changes influenced by exercise intensity and duration (Mulser & Moreau, 2023). For example, moderate aerobic exercise improves CBF more effectively than HIIT in young adults (Liu et al., 2023). Dynamic exercise increases vertebral artery flow more than carotid artery flow, highlighting distinct posterior and anterior cerebral circulation responses (Sato et al., 2011). Low to moderate‐intensity exercise also boosts hippocampal BDNF levels, promoting neurogenesis and enhancing cognitive function (Bhattacharya et al., 2023; Consorti et al., 2021; Daniela et al., 2022).

### 5.1 Exercise‐induced improvements in cerebrovascular reactivity

**FIGURE 2:**
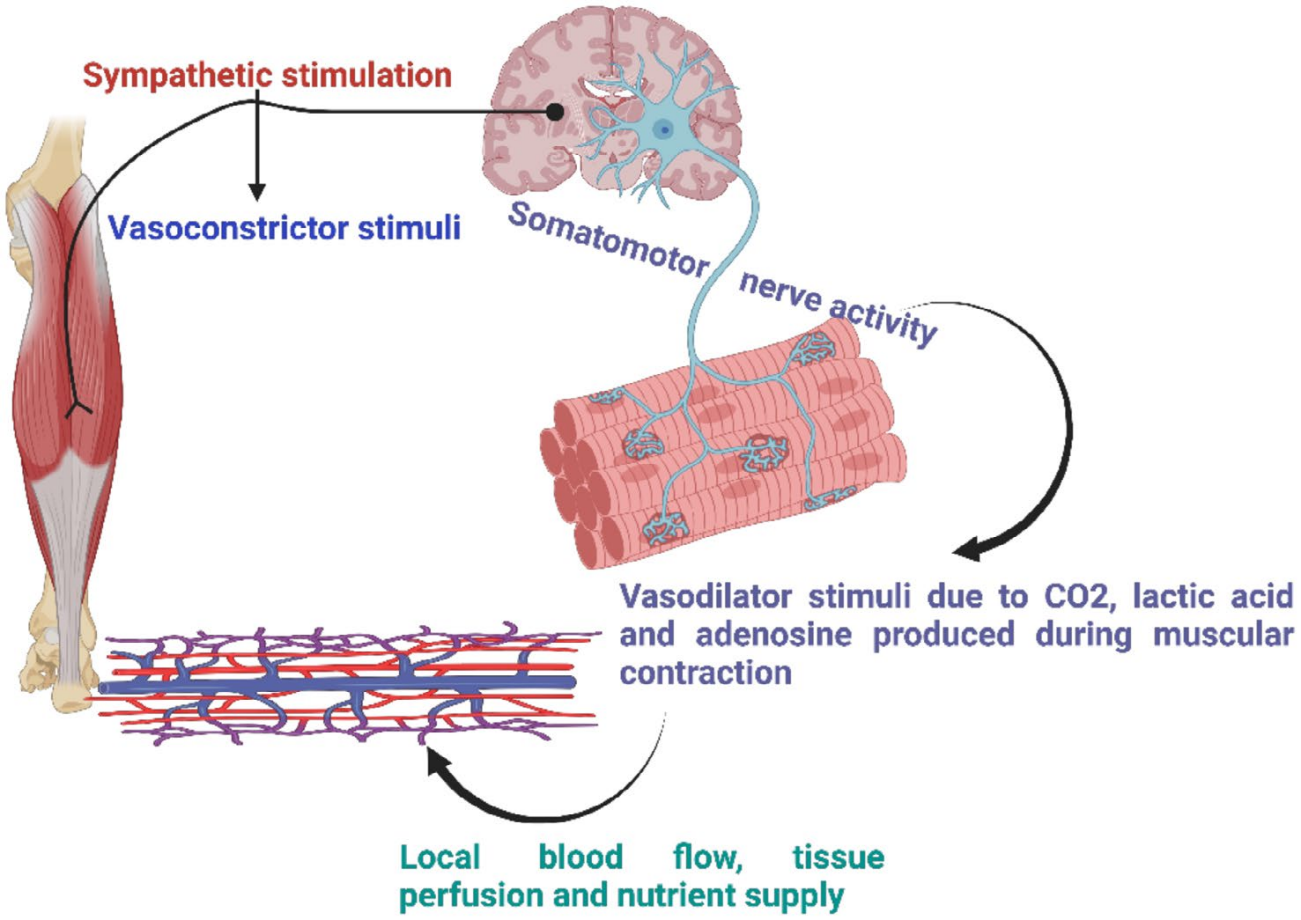
Neural control of metabolic demands during exercise.

The CBF regulation relies on cerebrovascular reactivity to systemic and local stimuli, especially arterial CO2 fluctuations. CO2, a potent vasodilator (Figure 2), increases CBF by promoting cerebral vasodilation, while impaired autoregulation correlates with higher CO2 levels (Claassen et al., 2021; de Sousa Fernandes et al., 2020). Cardiorespiratory fitness preserves cerebrovascular function, likely through aerobic exercise's benefits on cardiovascular and metabolic health (Tomoto & Zhang, 2024). During exercise, increased somatomotor activity induces skeletal muscle contraction and the release of vasodilatory metabolites like lactic acid and adenosine, leading to functional hyperemia (Claassen et al., 2021). The blood–brain barrier (BBB), comprising endothelial cells, mural cells, and astrocytic end‐feet, maintains cerebral homeostasis (Singh & Vellapandian, 2023) and is supported by endothelial perlecan‐based basement membrane and nitric oxide‐mediated vascular tone regulation (Lacoste et al., 2024). Gap junctions facilitate vascular smooth muscle cell hyperpolarization and arteriole dilation (Garland & Dora, 2021; Ottolini & Sonkusare, 2021). Exercise improves neurotransmitter dynamics and increases cortical neuron density (Bhattacharya et al., 2023). Regular moderate exercise enhances vascular reactivity by boosting nitric oxide production, mitigating oxidative stress (Tanaka et al., 2015), and promoting arterial remodeling to delay endothelial dysfunction and arterial stiffness (Song et al., 2022). Moreover, exercise strengthens BBB integrity (Małkiewicz et al., 2019) and promotes capillary growth and vascular relaxation through improved endothelial function (De Ciuceis et al., 2023). Given its role in enhancing cerebrovascular health, exercise training is a promising strategy for mitigating age‐related conditions such as stroke and Alzheimer's disease (AD) (Mcleod et al., 2019).

## 6 INFLUENCE OF EXERCISE ON THE BRAIN‐HEART AXIS IN NEURODEGENERATIVE DISEASES

Exercise mitigates neurodegenerative diseases by enhancing CBF and reducing cerebrovascular stiffness, critical factors linked to AD and cognitive decline (Korte et al., 2020; Park & Hwang, 2021). Moderate‐to‐severe intensity aerobic and resistance exercises are recognized as effective interventions to prevent or slow AD progression (Ribarič, 2022). Beyond improvements in VO2 max, strength training restores posture, motor coordination, and muscle mass, compensating for functional impairments common in AD patients (Bliss et al., 2023; Valenzuela et al., 2020). Exercise exhibits dose‐dependent relationship with delayed AD onset by reducing amyloid‐beta (Aβ) burden, enhancing motor performance, and mitigating depression (Wang et al., 2021). Mechanistically, driven by BDNF, exercise stimulates gray and white matter growth, neurogenesis, and neurovascular coupling (Andrade‐Guerrero et al., 2023; Colmenares et al., 2021). Furthermore, in AD models, reduced neurotrophin expression impairs neuronal survival and neurogenesis (Bonanni et al., 2022; Magaña et al., 2023). Regular aerobic training enhances cerebral perfusion, strengthens neuroplastic signaling pathways, and delays cognitive deterioration (Devanand et al., 2023; Huang et al., 2023).

In Parkinson's disease (PD), dopaminergic neuron loss in the ventral tegmental area and substantia nigra pars compacta impairs motor and cognitive function (Muddapu & Chakravarthy, 2021). Exercise enhances dopaminergic signaling and induces glial cell line‐derived neurotrophic factor (GDNF) expression, supporting synaptic integrity and motor recovery (Bispo et al., 2024; Ruiz‐Tejada et al., 2022). Through increased synaptogenesis, neurotransmitter modulation, and improved cerebral perfusion (Bhattacharya et al., 2023), exercise confers neuroprotection. As earlier noted, neurotrophic factors, including BDNF, IGF‐1, and VEGF, are crucial in maintaining brain health. However, early in PD, BDNF levels decline (Soke et al., 2021), suggesting its role in its progression. Regular exercise counters neurodegeneration by reducing oxidative stress, enhancing growth factor signaling, and stabilizing dopamine receptor function (Bonanni et al., 2022; Mahalakshmi et al., 2020). Vascular cognitive impairment (VCI), primarily driven by cerebral small vessel disease, is a major cause of dementia after AD. Aerobic exercise may slow VCI progression and reduce dementia risk (Hannawi, 2024; Huang et al., 2023; Van Der Flier et al., 2018; Ye et al., 2025).

## 7 BIOMARKERS OF NEUROVASCULAR AND CARDIOVASCULAR HEALTH

Biomarkers are essential for detecting physiological changes, diagnosing conditions, and guiding therapeutic interventions. In the context of exercise, biomarkers are crucial for assessing neurovascular and cardiovascular health (Trillaud et al., 2023). Exercise influences several biomarkers associated with these systems (Table 1). BDNF released from neurons supports neurogenesis (Figure 1), synaptic plasticity, and cognitive function, with levels increasing after both anaerobic and endurance training (Murawska‐Ciałowicz, Wiatr, et al., 2021; Sabita et al., 2023). Similarly, VEGF, which promotes angiogenesis and neuroprotection, shows elevated levels during exercise and is linked to reduced cognitive decline and increased hippocampal volume in Alzheimer's patients (Ahmad & Nawaz, 2022; Ribarič, 2022; Zarezadehmehrizi et al., 2021). Additionally, exercise activates the endocannabinoid system, with endocannabinoids like N‐arachidonoylethanolamine (AEA) and 2‐arachidonoylglycerol (2‐AG) supporting mood regulation, pain relief, and neuroprotection (Zou & Kumar, 2018). Cardiac biomarkers, such as cardiac troponin (cTn) and creatine kinase, may transiently rise following exercise, reflecting myocardial contraction and muscle stress (Aengevaeren et al., 2021; Li et al., 2022; Markus et al., 2021). Prolonged elevation in these markers may indicate cardiac or muscle injury (Okon et al., 2025). Myoglobin, a marker for myocardial ischemic injury, assists in evaluating training load and cardiac events, although its specificity remains debated (Okon, Nwachukwu, et al., 2024; Tilea et al., 2021). Regular moderate aerobic exercise increases BDNF and VEGF, improving cognition and neurogenesis, while elevated cTn, creatine kinase, and myoglobin suggest vascular damage (Aengevaeren et al., 2021; Soke et al., 2021). However, acute exercise can elevate BDNF and VEGF without affecting cardiac biomarkers (Bhattacharya et al., 2023). Understanding biomarker responses to exercise can enhance diagnostic accuracy and therapeutic strategies for neurocardiovascular health.

**TABLE 1:** Key biomarkers in neurocardiovascular adaptation.

Biomarker	Function	Exercise impact	Applicability
BDNF	Neurogenesis, synaptic plasticity	Increased with aerobic and HIIT	Depression
VEGF	Angiogenesis, vascular health	Enhanced by endurance training	Alzheimer's disease, depression
Endocannabinoids	Mood, autonomic modulation	Elevated post‐exercise	Depression
cTn	Myocardial stress indicator	Temporarily elevated in intense exercise	CVD
CK	Muscle damage marker	Peaks post‐exercise, aids recovery	AMI

Abbreviations: AMI, acute myocardial infarction; CVD, cardiovascular disorders.

## 8 PUBLIC HEALTH IMPLICATIONS OF BRAIN–HEART EXERCISE RESEARCH

Recent research has demonstrated that exercise improves the health of sedentary individuals, particularly those with neurovascular disorders (Bonanni et al., 2022). Most of them focus on the acute and transient effects of exercise, revealing its advantages for individuals with limited exercise. The mechanisms underlying the complex relationship between exercise and neurovascular function are supported by extensive scientific evidence, with advancements in neuroimaging providing crucial insights into these pathways (Ntikas et al., 2022). Regular physical activity, particularly aerobic exercise, has been shown to prevent and treat conditions such as hypertension, diabetes, PD, and anxiety, while improving vascular function (Gupta & Gupta, 2020; Srinivas et al., 2021). Exercise has been proven to enhance cognitive function and mitigate cardiovascular disorders in sedentary individuals, serving as an effective preventive and therapeutic strategy for neurovascular diseases (McDonnell et al., 2011). Furthermore, physical activity has a significant impact on academic performance, especially as neurophysiological processes linked to exercise can improve learning capacity and cognitive function in young populations (Ferreira Vorkapic et al., 2021). Regular aerobic exercise interventions are particularly beneficial in educational settings, including elementary, middle, and university institutions, helping students stay focused and perform better academically (Chen et al., 2022; Seger et al., 2022). Combining acute bouts of exercise with mental tasks can further enhance vascular and cognitive performance, making this approach valuable for academic and professional contexts (Cornelius et al., 2020).

### 8.1 Clinical applications of brain–heart connectivity in exercise interventions

Perioperative brain disorders caused by cardiac failure can lead to stroke, encephalopathy, and cognitive impairment (Chaudhary et al., 2024). These conditions can be mitigated through structured light to intense exercise therapy (Rondão et al., 2022). Post‐surgery, 30%–65% of patients experience cognitive dysfunction, with 20%– 40% continuing to experience it 5 months later, making it the most common neurological consequence (Rost et al., 2022). Exercise, particularly strength training, can reduce stroke‐related brain dysfunction (Shahid et al., 2023). Atherosclerosis, diabetes, systolic hypertension, advanced age, and previous stroke are risk factors that can be managed with regular exercise (Ciumărnean et al., 2021). Exercise interventions should be tailored to individual health status and incorporated into treatment plans for cardiovascular and neurodegenerative diseases, promoting brain–heart synergy and improving overall health outcomes (Catrambone & Valenza, 2021).

### 8.2 Recommendations for future research

Exercise interventions should be incorporated into treatment plans for cardiovascular and neurodegenerative diseases. Future studies should investigate exercise‐induced neurovascular coupling, its effects on cardiovascular regulation, and neurovascular adaptations in health promotion settings. Further research on molecular mechanisms underlying exercise‐induced adaptations in diseases like Parkinson's and Alzheimer's is needed. Though aerobic exercise is recognized as a therapeutic strategy, establishing universal guidelines for its preventive and therapeutic applications requires further investigation. As a limitation, this review lacked focus on specific populations and exercise types; future reviews should address these aspects.

## 9 CONCLUSION

Exercise plays a critical role in promoting both cardiovascular and brain health, with the brain–heart connection offering a compelling case for its integration into clinical practice. The neurophysiological pathways linking exercise to improved cognitive function and cardiovascular health are well‐documented, with benefits including enhanced neuroplasticity, improved cerebrovascular reactivity, and reduced cardiovascular risk factors. Regular exercise, particularly aerobic exercise, can mitigate the impact of neurodegenerative diseases and cardiovascular conditions. By understanding these mechanisms, healthcare providers can develop targeted interventions that foster brain–heart synergy, ultimately improving patient outcomes. Incorporating exercise into treatment plans, particularly for at‐risk populations, is crucial for optimizing overall health.

## AUTHOR CONTRIBUTIONS

GJU conceptualized, visualized, participated in writing the original draft, review and editing, and supervised the project; JGO, IAO, and OMA participated in writing the original draft, review and editing; AA, RDA, BO, NN, and EME participated in writing—review and editing the manuscript; OKY participated in writing—review and editing, and supervised the project. All authors have read and approved the final version of the manuscript and agree with the order of presentation of the authors.

## FUNDING INFORMATION

No funding information provided.

## CONFLICT OF INTEREST STATEMENT

The authors declare that they have no competing interests.

## Data Availability

Not applicable.
